# 

**Published:** 1888-06-16

**Authors:** 


The class of children who most need the care of the charitable is well illustrated by two pathetic incidents related by kindergarten teachers in San Francisco. One of them says: "Many of our little ones come in the morning with very little, if any breakfast. Not liking to impose too much on our kind janitress, we send and buy bread, and always tell the children if they are cold, hungry, or want anything, they must tell us. And our baby boy, with child-like faith, thought it would hold good in every thing, so one morning he came and said: 'I do wis I had one kitty; I'se nussin to pay wiz at my house but *wats*.'" The other writes: "One of my little girls invited me to go into the country with her, and as an inducement said, 'You shall have a nice ride.' Little Phil, not to be outdone, said: 'I had a nice ride last week!' I said, "Did you, dear? I am glad." He answered: 'Baby's dead, and I was tooked to the funeral.' Poor children! how sad that the greatest pleasure they know, outside of the kindergarten, should come to them through the death of 'the baby.'"

### The Dispensers' Column.

An instance of the extraordinary explosive power of a small quantity of nitro-glycerine is recorded by Dr. Gorup Besanez. The accident was the explosion of only ten drops of the substance in his laboratory, and the astonishing effects he records as resulting from this explosion are well calculated to give a most respectable and respectful notion of the powers of nitro-glycerine. One of the doctor's pupils in the course of an investigation placed the above-mentioned quantity of the substance in question in a small cast-iron dish heated over a small Bunsen gas-burner in common use in laboratories. While so engaged the nitro-glycerine exploded with extreme violence, breaking forty-six panes of glass in the window of the laboratory; hurled the iron dish against the brick wall, the iron stand upon which it was supported being split and partly twisted out of shape, and the tube of the Bunsen burner split and flattened. Those in the laboratory fortunately escaped without injury.

**Special Notice to Newsagents, Advertisers, and the Trade.**

**CHANGE OF OFFICE AND PUBLISHER.**

Notice is hereby given that the Office of The Hospital will henceforth be at 38, Old Jewry (Cheapside end), E.C., to which address all communications should be sent. Mr. A. Doig has been appointed Advertising Agent, and Mr. J. H. S. Hanning, Manager. All Cheques and Post Office Orders should be made payable to J. H. S. Hanning, 38, Old Jewry, E.C. crossed Lloyds, Barnetts, and Bosanquets Bank (Limited).

**To Secretaries and Matrons.**

The Editor will be glad to have a copy of the Regulations, and also of every Report issued from the Press of Asylums, Hospitals, Convalescent and Nursing Institutions, as well as those of other Charities, so soon as they are published, and an early copy in duplicate of all appeals for funds. Notices of Annual and other Meetings, Festival Dinners, etc., will be welcomed. All such communications should be addressed to *The Editor, The Lodge, Porchester Square, London, W.* Any superintendent, secretary, matron, or other chief official who will regularly send such documents and information will be registered to receive free of cost a cover for binding The Hospital in half-yearly volumes on sending name and address to the publisher.

### Amusements with Profit for all Ages.

### FOR MEN AND WOMEN.

### First Quarterly Poetical Competition.

Commenced May 26th, 1888. Ends August 25th, 1888.

This quarter a PRIZE of ONE GUINEA will be given to the competitor who gains the highest number of marks for original poems, on any subjects suitable for insertion in this paper. All contributions sent in will be credited according to merit eighteen marks being the highest score for any one contribution. This competition to alternate weekly with the set acrostic competition.

### Acrostic Competition.

This quarter the original acrostic competition will be discontinued, and A PRIZE of HALF-A-GUINEA will be given to the competitor who gains the highest score for solution of acrostics set. *One mark* only will be given to the competitors who solve all the lights of each acrostic.

Exception will be made in the case of *quotation acrostics*, when *two marks* will be given, *one* for the correct solution of all *lights*, and *one* mark for the *source* of the quotations.

This week credit will be given for

**No. 2.—Original Poem.**

### Results of No. 1.—Original Poem.

Dodo (14), Padre (14), K. M. (13), A. M. E. (12), Patience (11), Hesperornis (10), Mater (10).

### THE WORD COMPETITION.

Third Quarterly Competition commenced
May 5th, 1888; ends August 4th, 1888.

Three prizes of one guinea, 15*s.* and 10*s.* 6*d.* will be given each quarter to the three competitors who obtain the highest number of marks by making the largest number of words derived from the word or phrase set for anatomical dissection; that for this, the *sixth* week of the quarter, being

"The Argosy."

Observe.—Proper names, abbreviations, foreign words, words of less than four letters, and repetitions are barred; plurals, and past and present participles of verbs are allowed. Nuttall's *Standard* dictionary only to be used.

N.B.—All words sent in must be arranged *alphabetically*, with correct total affixed.

### Results of Fourth Week.

*Oak Apple.*—Reldas (27), Tinie (26), Dollie (26), Puff (26), Patience (26), Lightowlers (25), Lauriston (25), Sym (24), Carmen (23), Dodo (23), Ion (23).

### Notices to Correspondents.

*Edge.*—The Word Competition cannot be cr edited unless sent in *weekly*.

### THE CHILDREN'S CORNER.

PRIZES.
FOR MOST CORRECT ANSWERS TO PUZZLES.
This Commenced October 1st, 1887.
One Pound a Month.
A PRIZE OF THREE GUINEAS

to the Competitor who shall have sent in the largest number of correct or best answers during the twelve months.

### Puzzles.—Second Week.

No. 6. If four towns' initials and one county you name,
They will give you the name of a warrior of fame;
And then, when their finals are carefully scanned,
They will show you a river in the south of England.

1. A town in Lincolnshire. 2. A town and seaport in Russia. 3. An Irish county. 4. A town in Italy. 5. An English cathedral city.

No. 7. Decapitation. I am a small animal; behead me and I am a river; behead me again and I become a verb.

No. 8. Floral Anagrams. 1. Men crush my hat. 2. A live lion, not a pet. 3. Neat chairs.

No. 9. Missing Letter Puzzle.

U—d—r—h—g—e—n—o—d—r—e

W—o—o—e—t—l—e—i—h—e

A—d—u—e—i—m—r—y—o—e

U—t—t—e—w—e—b—r—s—h—o—t

C—m—h—t—e—c—m—h—t—e—,c—m—h—t—e—

H—r—s—a—l—e—e—

N—e—e—y———B—t—i—t—r—n—r—u—h—e—t—e—.

No 10. Standard gold is worth £3 17*s.* 10½*d.* an ounce. What is the weight of the smallest mass from which an exact number of sovereigns could be coined?

### Results of Fourth Week Puzzles.

No. 13. Geographical Acrostic.

T hebe S
U sur P
R eggi O
K herso N
E ig G
Y onn E

No. 14 Picture Puzzle. On S T is the best Poll I see=" Honesty is the best policy."

No. 15. Biographical Charade. Ben-net=Bennet.

No. 16. Enigma. A sigh.

*All right.*—A. C. K, Scrooge. *Three right.*—The Young Canadian. *Two right.*—Nunkey, Plummy Fluff, Spider, Gem, Geranium. *One right.*—Alsie.

### Results of Eighth Monthly Competition.

*A. C. K* and *Scrooge*, having already gained prizes since this Competition commenced in October last, are disqualified from taking another; we therefore award the Prize of One Pound to *Nunkey* (Master Archd. McIntyre, 87, Grosvenor Road, Pimlico, S.W.; aged 14).

### The Student's Column.

The following are the questions set by the Society of Apothecaries of London at their last examination for the L.S.A. diploma, on the 14th May, 1888:

### EXAMINATION IN MEDICINE.

Medicine (four questions only to be answered).—1. How would you examine a patient who complained of Palpitation? State what treatment you would adopt in the cases you mention. 2. Indicate the physical signs that may be found in the various stages of pulmonary consumption. 3. What points would you look to in a case of hemiplegia? 4. How would you proceed to examine a patient who presented signs of obstruction of the bowels? 5. Give the physical signs of aortic regurgitation; and state what treatment you would adopt. 6. Describe a case of rickets in a child eighteen months of age.

Therapeutics (one question only to be answered).—1. What treatment may be used to subdue cough in various cases as met with in practice? 2. How would you treat chronic constipation?

Pathology (two questions only to be answered).—1. Give the pathology of granular kidney. 2. Describe the pathology of the different stages of pneumonia 3. What is meant by embolism? Under what conditions does embolism occur?

### EXAMINATION IN SURGERY.

Surgery (four questions only to be answered).—1. Give the causes, symptoms, and treatment of fracture of the ribs, and mention some of the most usual complications, and the way you would deal with them. 2. Give the causes, diagnosis, and treatment of suppuration in the female breast. 3. Give the symptoms, treatment, and complications of gonorrhœa. 4. Under what circumstances might venesection be required? Give a detailed description of the operation. 5. Give the symptoms and treatment of acute laryngitis in its various stages.

Surgical Pathology (one question only to be answered).—1. Mention the different varieties of sarcomata, and state where they are most frequently met with. 2. Describe the mode of formation of an acute abscess.

Surgical Anatomy (one question only to be answered).—1. Give the relations of the tonsil. 2. Mention in their order the parts cut through in amputation at the lower third of the fore-arm.

### EXAMINATION IN MEDICINE

Forensic Medicine and Hygiene (three questions only to be answered)-—1. What are the symptoms, treatment, and post mortem appearances in a case of Hydrate of Chloral poisoning? 2. Discuss the objections to the Hydrostatic test as an evidence of Live Birth. 3. What are the symptoms produced from Lead contamination of Water? What quantity would produce poisonous symptoms? And how would you detect the presence of Lead in Water? 4. Describe in detail the symptoms of a case of general Paralysis of the Insane.

### EXAMINATION IN MIDWIFERY.

Midwifery (three questions only to be answered).—1. What are the causes of Uterine Inertia? How would you diagnose the condition? And how treat it under the various circumstances? 2. Give the diagnosis and management of breech presentations. 3. Describe fully the mechanism of labour in a right mento-posterior face presentation. 4 Describe the diagnosis of a "simple flat" pelvis, and give the mode of delivery suitable in various degrees of contraction in such pelves.

Gynæcology (one question only to be answered).—1. Describe the varieties of uterine polypi, and give the symptoms, diagnosis, and treatment. 2. What are the indications for dilating the Cervix in the non-pregnant condition? Describe in detail the method you would adopt.

Yellow hair, though now somewhat out of fashion, has often been esteemed a mark of great beauty. The Venetian ladies used to bleach their hair by stretching it over crownless broad-brimmed hats and sitting in the sun; and in the time of the Plantagenets saffron was used as a dye to produce the desired colour. Queen Elizabeth made yellow hair, and small waists also, fashionable. We are told that this dress loving monarch had her yellow locks "fair attired with rich strings of golden wire, and gold wire was abouten all her genteel middle small."

